# The impact of characteristic factors of the direct-to-consumer marketing model on consumer loyalty in the digital intermediary era

**DOI:** 10.3389/fpsyg.2024.1347588

**Published:** 2024-03-04

**Authors:** Weizhe Mu, Yating Yi

**Affiliations:** School of Management, Harbin University of Commerce, Harbin, China

**Keywords:** digital intermediary era, direct-to-consumer marketing model, perceived value, stimulus-organism-response, consumer loyalty

## Abstract

In the era of digital intermediaries, the direct-to-consumer (DTC) marketing model is gaining prominence in the retail and brand marketing domains. This model exhibits a distinct advantage over traditional models in cultivating loyalty. Consequently, this study employs a survey-based approach and utilizes the Stimulus-Organism-Response (SOR) theory to construct a structural equation model and investigate the relationship between the DTC marketing model’s characteristic factors and consumer loyalty. The results indicate that cost-effectiveness and social media marketing directly and positively influence consumer loyalty, while product features indirectly contribute to loyalty through perceived emotional value. Multi-channel integration indirectly influences loyalty through perceived functional value. Additionally, the varying degrees of influence highlight social media marketing as the most impactful factor and product features as the least influential. The research findings underscore the importance of strengthening social media marketing, optimizing product features, reducing information asymmetry, and integrating multiple channels to enhance consumer loyalty. This study enriches the understanding of the DTC theoretical framework in the field of marketing and provides new perspectives for formulating marketing strategies.

## 1 Introduction

In the field of marketing, the characteristics of the digital intermediary era are becoming increasingly prominent due to the widespread adoption of the Internet and the rapid development of technologies. Such technologies include big data and artificial intelligence, as well as digital platforms like social media (Gawer, 2022). Defined by Chircu and Kauffman (2000) as distinct from traditional intermediaries that match suppliers and customers in established markets, digital intermediaries are influenced by the contemporary impact of the Internet. In their definition, Kleis Nielsen and Ganter (2018) also include digital intermediaries using various digital tools—e.g., digital software, technologies, and devices—to deliver providers’ content to consumers. However, the definition of digital intermediaries continues to refine and expand. For example, González-Tosat and Sádaba-Chalezquer (2021) suggest that digital intermediaries may not belong to the media industry, although their products and services are related to the media production chain and enrich media products and value addition. Therefore, this paper defines the digital intermediary era as a period in which businesses—in the context of Internet ubiquity and technological advancement—extensively utilize various digital tools (such as digitalized intermediary platforms including social media) to establish connections with consumers and engage in digital marketing activities.

The development of the digital intermediary era has brought about significant changes in the marketing environment and approaches. The direct-to-consumer (DTC) marketing model is a popular research tool in the field of brand marketing, where brand owners demand innovative modes. The DTC marketing model can be used when brands sell directly to consumers, bypassing any third-party independent retailers, wholesalers, or other intermediaries (Gielens and Steenkamp, 2019). Key characteristics of the DTC marketing model include consumer-centricity, direct interaction with consumers, high autonomy and specialization, product segmentation with an emphasis on design and functionality, cost-effectiveness, multi-channel integration, and a focus on social media marketing. According to Jin and Shin (2020), brands adopting this marketing model leverage digital channels for marketing and sales, distinguishing themselves from traditional models through both specialization in individual or small series of related products and innovation in products or business models. Rangan et al. (2021) assert that the DTC marketing model is popular among companies as well as consumers. Particularly in the fiercely competitive retail sector, many brands are adopting the DTC marketing model.

In response to these changes in the retail sector, the DTC marketing model is a current research hotspot. Andersson et al. (2020) employed qualitative research methods to investigate how DTC companies implement their online brand strategies. They found that social media plays a pivotal role in the online brand strategies of DTC enterprises. To examine the impact on consumer psychology and behavior, Paintsil (2019) explored how consumers engage in modern luxury DTC brand marketing activities on social media. The study delved into the cognitive, emotional, behavioral, and offline factors influencing consumer engagement with modern luxury DTC brands on social media. Kim et al. (2021), through in-depth interviews, identified factors such as co-creation, cost-effectiveness, website attractiveness, brand uniqueness, social media engagement, and brand innovativeness that significantly influence consumer attitudes and repurchase intentions toward DTC brands. Hillerborn and Eriksson (2022) found that social media, product knowledge, customer service, integrity, website manageability, and unique value addition are essential factors for enhancing consumer experience in the DTC marketing model. Additionally, Prasad and Ghosal (2022) investigated transaction security in online purchasing behavior, availability of innovative products, and product quality to provide algorithmic solutions for the development of DTC marketing models. Li and Sun (2023) demonstrated that data model algorithms permeate the entire business process of a company, improving the accuracy of personalized recommendation systems, the sustainability of subscription services, and efficiency of logistics optimization. Their study facilitates innovation and digital transformation for clothing DTC enterprises through e-commerce platforms. Schacker and Stanoevska-Slabeva (2023) developed a morphological box for a DTC model as a tool for business managers to find configurations that suit their operations.

In summary, previous studies explored various aspects of the DTC marketing model; however, research in this field remains in its nascent stage. The current focus on conceptual definitions, case analyses, and qualitative analyses lacks a comprehensive exploration of the characteristic factors of the DTC model. Furthermore, there are few empirical studies on the relationship between the DTC marketing model and consumer loyalty. The specific factors that positively influence consumer loyalty have not been investigated in detail and the underlying mechanisms remain unclear. Additionally, practical and detailed guidance for DTC brand loyalty and long-term development is currently lacking. Therefore, to enhance consumer perceived value effectively and cultivate loyalty, it is crucial to conduct in-depth research on the impact of DTC marketing model characteristic factors on consumer loyalty. This study examines:

1. The direct impact of DTC marketing model characteristic factors on consumer perceived (functional, emotional) value and loyalty.
2. The mediating roles of perceived functional value and perceived emotional value in the relationship between DTC marketing model characteristic factors and loyalty.
3. The varying weights of different characteristic factors in influencing loyalty.

The aims are to supplement and expand the application of relevant theories in the field of DTC marketing, and assist businesses and brand owners to establish clear objectives, obtain precise positioning, leverage distinctive features, and achieve substantial development.

Section 2 of this paper delves into the characteristic factors of the DTC marketing model through brand cases and relevant literature. The 4Ps and 4Cs theories are used to categorize four characteristic factors, and theories on perceived value and loyalty, and the SOR theory, are reviewed. Section 3 outlines the proposed research hypotheses and constructs the research model. Section 4 introduces the research methodology, covering scale sources, questionnaire design, data collection, and reliability, validity, and bias tests. Section 5 describes how a structural equation model was constructed, and tests on direct effects, mediating effects, and variable weights were conducted. This is followed by an analysis of the results. Finally, Section 6 summarizes the findings, presents relevant recommendations, analyzes the theoretical contributions and practical implications of the study, and discusses research limitations and future directions.

## 2 Literature review and theoretical background

### 2.1 Exploration of DTC marketing model characteristics

The term DTC originated in the 1990s and referred to an online business model where pharmaceutical companies directly marketed to consumers (Donohue, 2006). In recent years, influenced by the development of digital intermediaries (such as social media) and the impact of COVID-19, DTC has been used in the consumer-goods retail industry to denote a disruptive marketing model and brand strategy that bypasses traditional intermediaries and directly targets consumers (Schlesinger et al., 2020). The DTC marketing model is increasingly popular among retail brands. Although current DTC brands have common features, they also display distinctive model characteristics that are aligned with their respective brand features.

In terms of product innovation, the DTC footwear brand Allbirds incorporates a high-tech concept into its products, introducing unique and novel machine-washable wool sneakers that have garnered favor from a considerable consumer base, including former U.S. President Barack Obama (Matsuhisa and Matsubayashi, 2024). Regarding pricing strategy, the DTC apparel brand Everlane has gained industry attention by creatively disclosing cost information throughout its supply chain, including design, production, marketing, and transportation, demonstrating radical transparency and providing consumers with a sense of ultimate value for money (Gerlick, 2019). Warby Parker, a DTC fashion-eyewear brand, disrupts traditional offline eyeglass fitting practices by offering efficient and convenient online services, creating an outstanding consumer experience with low-cost, high-quality products that have quickly outpaced some traditional eyewear brands (Berman et al., 2016). In terms of distribution channels, the DTC beverage brand Genki Forest integrates online and offline channels, efficiently collecting customer information and feedback, allowing it to quickly establish a presence among numerous traditional beverage brands (Sun, 2022). Regarding marketing strategy, Perfect Diary, a rising star in the DTC beauty industry in China, seamlessly connects various online and offline channels, providing a complete service chain from online ordering to offline experiences. Through various social media marketing techniques, Perfect Diary engages in in-depth communication with consumers, fostering frequent interactions and building lasting and solid connections (Lu, 2023). In addition, Chinese-style brand Huaxizi incorporates traditional Chinese culture and craftsmanship into modern cosmetics research and manufacturing. It utilizes social media platforms such as WeChat, TikTok, and Xiaohongshu for refined and precise user operations and personalized marketing (Tang, 2023).

The above brands, each leveraging different advantages of DTC marketing models, have gained prominence on the international stage, attracting significant consumer attention and loyalty.

### 2.2 The 4Ps and 4Cs in DTC marketing

Based on the above brand cases, to establish a comprehensive combination of DTC marketing models, this study utilizes Kotler (1967) 4Ps marketing mix theory of “Product, Price, Place, Promotion” and combines it with Lauterborn’s (1990) customer-centric 4Cs theory of “Customer’s needs and expectations, Customer’s cost, Customer convenience in purchasing, and Customer communication with the enterprise.” Although the development of the digital intermediary era and changes in the marketing environment requires the theory to be modified, its use as a framework for marketing model research is still applicable. The changes in the digital environment can be incorporated into the model by modifying the “P” and “C” elements (Constantinides, 2006).

In the digital intermediary era, the aspects of “product, price, place, and promotion” can be redefined and redesigned. Combining the marketing environment with DTC brand cases, we can summarize the combination of characteristic factors in the DTC marketing model. In terms of product, the diverse and variable consumer base requires brands to be consumer-oriented, provide unique and innovative products and services based on personalized consumer needs, and offer consumers value beyond the products and services themselves (Tanrikulu, 2021). Combining the unique, novel, and high-tech features present in the DTC brands, we derive the product factor in the DTC marketing model: Product Features (Product Uniqueness and Product Innovativeness). In terms of price, with the involvement of digital intermediaries, the asymmetry of information between consumers and businesses has been reduced, leading to increased transparency (Helberger et al., 2018). Consumers no longer only compare gains and losses in monetary terms but they measure the value of what they pay in terms of money, effort, and time against the product’s processes, features, quality (Kumar and Reinartz, 2016). Corresponding to this change, several DTC brand cases have shown high transparency, creating low-cost, high-quality, and high-value products. Therefore, we categorize the pricing factor in the DTC marketing model as: Cost-effectiveness.

In terms of channel, businesses are not only required to create new ways to reach customers but they also need a synergy between online and offline channels. By integrating multiple channels, they can provide consumers with a seamless shopping experience (Herhausen et al., 2015). To cater to this trend, DTC brands—in addition to operating online channels—have started to expand offline channels by establishing flagship stores, experiential stores, and pop-up shops. Leveraging the advantage of bypassing traditional intermediaries, DTC brands control multiple channels to achieve online and offline coordination. Thus, we derive the channel factor in the DTC marketing model: Multi-channel Integration. In terms of promotion, businesses are required to engage in active and effective two-way communication with consumers using digital tools to establish lasting connections, and obtain timely two-way feedback, create shared value; processes which can have mutual benefits (Paintsil and Kim, 2022). DTC brands, leveraging their digital-native characteristics, fully utilize various social media tools for marketing to gain widespread attention in the marketing industry. Therefore, we categorize the promotion factor in the DTC marketing model as: Social Media Marketing.

### 2.3 Stimulus-organism-response (SOR) theory

Because consumer loyalty is fostered through multiple pathways, this paper employs SOR theory (Mehrabian and Russell, 1974) to illustrate the complex relationships between customer perceptions and loyalty behaviors. SOR can delineate the processes of individual decision-making and responses. Stimuli, as driving factors, influence individuals’ internal psychological and external reactive processes. Factors such as the environment, product, price, and service are common stimuli (Lin and Shen, 2023). The organism represents an internal cognition and emotional perception between stimuli and responses (Leone et al., 2005). Factors such as perceived value, perceived risk, and satisfaction are typically studied as organism elements. Response involves internal positive or negative attitudes, external approach or avoidance behaviors due to external stimuli, and internal psychological perceptual processes. Purchase intention and loyalty are response factors (Thomas and Mathew, 2018). Thus, within the SOR theoretical framework, the four characteristic factors of the DTC marketing model are considered as stimuli, perceived value as an organism factor, and consumer loyalty as a response factor.

#### 2.3.1 DTC marketing model characteristic factors (S)

To ensure the DTC marketing model characteristics are examined in full, the distinct dimensions of each factor are measured. Product Features (Uniqueness and Innovativeness); Cost-Effectiveness; Multi-Channel Integration (Service Construct Transparency, Channel Choice Freedom, Content Consistency, Process Consistency); and Social Media Marketing (Informativeness, Relevance, Interactivity) are considered as independent variables.

#### 2.3.2 Perceived value (O)

Consumer perceived value is a critical factor in organizational strategic management. Although some scholars view perceived value as unidimensional, balancing gains and sacrifices (Morar, 2013), others argue for a multidimensional view. Sweeney and Soutar (2001) developed a scale with four dimensions: functional value, emotional value, monetary value, and social value. Multidimensional perceived value theory is an adaptable approach that does not require all its dimensions to be included (Hur et al., 2013). The dimensions of functional value and emotional value are used as mediating variables in this study because consumers’ loyalty toward DTC brands is influenced by utilitarian and hedonic components (Sameeni et al., 2022).

#### 2.3.3 Consumer loyalty (R)

Maintaining existing customers incurs fewer costs in terms of time, effort, money, and resources compared to acquiring new customers. Loyal consumers engage in positive behavioral intentions including repeat purchases and word-of-mouth recommendations. Marketing practitioners and researchers recognize the importance of enhancing consumer loyalty for the long-term development of a business. Consumer loyalty theory (Oliver, 1999) states that consumer loyalty, manifested by a preference for a specific business or brand, is accompanied by a deep commitment to repeatedly purchase one company’s products. Consumer loyalty is used as the dependent variable in this study.

## 3 Research hypotheses and research model

### 3.1 Impact of DTC marketing on perceived value and loyalty

3.1

#### 3.1.1 Impact of product features on perceived value and loyalty

The current primary consumer groups of Millennials and Generation Z are characterized by their love of technology and interest in new products. They are non-conformist, seek personal uniqueness, and prefer expressing themselves in individualistic ways. This consumer demographic has developed esthetic fatigue toward traditional brands. They crave distinctive, creative products (Munsch, 2021) to highlight their personalities, identities, and unique tastes. If a brand can represent their interests and tastes, they can create brand loyalty in this demographic (Pappu and Quester, 2016).

DTC brands strive to cater to the needs of such consumers. Therefore, showing that they are in line with what modern consumers prefer is a key principle of their marketing strategy. Niche positions can be established by unique brand images, as well as innovative products with high-tech design-centric features (Li and Zhang, 2019). Product uniqueness refers to the extent to which a brand’s product differs from those of competing brands. Prominent distinctive features allow consumers to quickly notice, recall, and identify a brand (Foroudi et al., 2018). Product innovativeness—a brand’s ability to create new products or improve existing features to meet consumers’ emerging needs (Kuncoro and Suriani, 2018)—is manifested in their product attributes, service methods, marketing strategies, brand positioning, and market behavioral dynamics. Both product features of uniqueness and innovativeness can attract consumers and foster loyalty, providing DTC brands with a competitive advantage (He and Zhang, 2022).

Product features have a positive impact on consumers’ perceived functional value, leading to positive attitudes (Yu and Lee, 2019). When consumers perceive the purchased product as unique, their perceived emotional value increases (Zhang et al., 2020) increasing loyalty and repurchase intentions (Wang et al., 2019). Therefore, the following hypotheses are proposed:

*H1a*: Product features have a significant positive impact on consumers' perceived functional value of DTC brands.

*H1b*: Product features have a significant positive impact on consumers' perceived emotional value of DTC brands.

*H1c*: Product features have a significant positive impact on consumers' loyalty towards DTC brands.

#### 3.1.2 Impact of cost-effectiveness on perceived value and loyalty

Many DTC brands directly open up the supply chain source and production process to consumers. For example, some brand manufacturers live stream their production techniques of TikTok. Products are ordered on the platform and shipped directly from the factory to customers, bypassing many intermediate processes and costs. This approach offers quick, efficient, and reliable products at reasonable prices. Younger consumers are becoming more rational when selecting products, not necessarily focusing on high-end brands. Instead, they consider the cost-effectiveness of products, emphasizing the perceived cost: benefit ratio (Cakranegara et al., 2022).

Cost-effectiveness means that consumers perceive the value of the product as worth or exceeding the cost invested (Chaudhary et al., 2022). Typically, after consuming a product or service, consumers psychologically evaluate the money, effort, and other resources invested versus the functional utility and emotional value obtained (Chiu et al., 2014). The process may compare individual products and also different brands. When products from traditional brands are available at comparable prices and quality levels, consumers may view DTC brand products as having higher cost-effectiveness, thereby achieving higher perceived functional and emotional value (Lin et al., 2020). Kim et al. (2021) found that the cost-effectiveness factor in DTC brands positively influences consumers’ attitudes and loyalty behaviors such as repurchase intentions. Therefore, the following relationships are posited:

*H2a*: Cost-effectiveness has a significant positive impact on consumers' perceived functional value of DTC brands.

*H2b*: Cost-effectiveness has a significant positive impact on consumers' perceived emotional value of DTC brands.

*H2c*: Cost-effectiveness has a significant positive impact on consumers' loyalty towards DTC brands.

#### 3.1.3 Impact of multi-channel integration on perceived value and loyalty

In China, DTC brands utilize various online marketing channels, including official online stores, flagship stores on e-commerce platforms, social media platforms such as Weibo, TikTok influencer live streams, WeChat official accounts, and platforms like Xiaohongshu. Offline channels involve exclusive sales through brand flagship stores, experiential stores, pop-up shops, and shelves in supermarkets. Mature DTC brands adopt a combined online and offline marketing approach, effectively integrating different channels. The DTC marketing model allows DTC brand manufacturers to easily retain control over each channel, producing higher profits (Kim and Chun, 2018). In the digital era, although many consumers prefer utilizing online channels, Millennials and Generation Z consumers also enjoy offline shopping experiences (Alexander and Kent, 2022). Venkatesan et al. (2007) suggest that brands offering multiple channels are more attractive to consumers, increasing purchase frequency. Additionally, multi-channel brands with synergies between online and offline channels can enhance the shopping experience, providing consumers with higher perceived functional and emotional value; they therefore have a competitive advantage (Herhausen et al., 2015). Hence, investigating how multi-channel integration influences consumer loyalty and other behavioral responses is crucial (Xin et al., 2022).

Sousa and Voss (2006) measure multi-channel integration quality through four dimensions: service construct transparency, channel choice freedom, content consistency, and process consistency. Service construct transparency refers to consumers’ awareness and understanding of the various channels provided by the brand. Channel choice freedom allows consumers to freely select different channels to meet their needs when purchasing goods. Content consistency refers to the perceived consistency of information about products, prices, and promotions across different channels. Process consistency encompasses the consistency of various channels in terms of image, level, and timeliness of service. In this context, multi-channel integration positively influences consumers’ perceived functional and emotional value (Chen et al., 2020) and increases brand loyalty (Lazaris et al., 2021). Therefore, the following relationships are hypothesized:

*H3a*: Multi-channel integration has a significant positive impact on consumers' perceived functional value of DTC brands.

*H3b*: Multi-channel integration has a significant positive impact on consumers' perceived emotional value of DTC brands.

*H3c*: Multi-channel integration has a significant positive impact on consumers' loyalty towards DTC brands.

#### 3.1.4 Impact of social media marketing on perceived value and loyalty

The lives of Millennials and Generation Z are saturated by the Internet and digital products. They favor brands endorsed by celebrities, seek advice from social media influencers, and often use social media to search for brand-related information and stay informed about corporate dynamics (Hanifawati et al., 2019). DTC brands adeptly leverage this consumer characteristic by extensively utilizing social media for marketing activities. For instance, Glossier rapidly expanded by actively engaging with consumers through platforms like Instagram, Twitter, and Facebook, gathering consumer feedback, and continually developing and updating more appealing products (Sherman, 2016). Social media marketing involves digital intermediaries like social media to disseminate various brand-related information, promote products and services, and maintain interactive and mutually beneficial relationships with consumers (Hung and Khoa, 2022). The effectiveness of social media marketing can be measured using riskiness, personalization, usability, timeliness, novelty, informativeness, entertainment value, relevance, and interactivity (Hanaysha, 2022). Building on Alalwan’s (2018) study and considering the characteristics of DTC marketing and DTC brand consumers, this study selects the dimensions of informativeness, relevance, and interactivity to investigate the social media marketing characteristics of the DTC marketing model.

DTC brands allocate significant resources to maximize their social media marketing strategies and ensure their brand information is prominently presented. The presented information influences consumers’ perceived functional value (Ryu and Park, 2020). Personalized recommendations based on large amounts of customer data ensure that consumers receive content aligned with their interests to positively impact their perceived emotional value (Wang et al., 2021). Furthermore, the interactivity of social media marketing encourages consumers to actively participate in brand interactions by liking, commenting, sharing, and providing timely feedback. This fosters a close relationship between consumers and the brand and contributes to the co-creation of value (Jiang et al., 2021). Given that social media marketing has a positive impact on consumer loyalty (Ebrahim, 2020) and can enhance brand loyalty (Suharto et al., 2022), the following hypotheses are proposed:

*H4a*: Social media marketing has a significant positive impact on consumers' perceived functional value of DTC brands.

*H4b*: Social media marketing has a significant positive impact on consumers' perceived emotional value of DTC brands.

*H4c*: Social media marketing has a significant positive impact on consumers' loyalty towards DTC brands.

### 3.2 Impact of perceived value on consumer loyalty

Although some consumers evaluate the characteristics of businesses or brands rationally, others may value experiential aspects and purchase goods for pleasurable experiences (Schmitt, 1999). Building on these two perspectives, Lee and Overby (2004) identified two types of shopping values that influence consumer behavior: utilitarian and hedonic. In reality, consumer purchasing decisions or behavioral responses to a particular brand or product are often the result of the combined influence of rational and emotional factors. Consumers not only focus on functional values such as utility and cost-effectiveness but also consider emotional values related to interest and entertainment. The four characteristic factors of the DTC marketing model encompass both utility and enjoyment, providing consumers with two dimensions of perceived value. These factors influence consumer behavior aspects such as satisfaction, purchase intention, and loyalty through two pathways: perceived functional value and perceived emotional value. Thus, perceived value is the most crucial predictor of consumer intention to exhibit brand loyalty. Perceived functional and emotional values positively impact consumer loyalty, driving the development of brand loyalty (Lou and Xie, 2021). Therefore, the following hypotheses are proposed:

*H5a*: Perceived functional value for DTC brand consumers has a significant positive impact on loyalty.

*H5b*: Perceived emotional value for DTC brand consumers has a significant positive impact on loyalty.

### 3.3 Mediating role of perceived value

#### 3.3.1 Mediating role of perceived functional value

Creating perceived functional value for consumers is a crucial link in the brand value chain and a key factor in gaining a competitive advantage. It is closely related to cultivating consumer loyalty and promoting the long-term development of the brand (Gupta et al., 2020). Factors such as service quality are crucial antecedents of perceived functional value (Molinillo et al., 2021), which is in turn, an essential driver of consumer loyalty. Perceived functional value is used as a mediating factor to represent the psychological processes between external stimuli and customer loyalty response as it is positively correlated with continuous purchase intention (Luo et al., 2023). Because the relationship between personalized offerings and continuous purchase intention is mediated by perceived functional value, the following hypotheses are proposed:

*H6a*: Perceived functional value for DTC brand consumers significantly mediates the impact of product features on loyalty.

*H7a*: Perceived functional value of DTC brand consumers significantly mediates the impact of cost-effectiveness on loyalty.

*H8a*: Perceived functional value for DTC brand consumers significantly mediates the impact of multi-channel integration on loyalty.

*H9a*: Perceived functional value for DTC brand consumers significantly mediates the impact of social media marketing on loyalty.

#### 3.3.2 Mediating role of perceived emotional value

In the DTC marketing model, brand merchants enhance consumers’ perceived emotional value by making their products novel, interesting, cost-effective, easily accessible through various channels, and incorporating entertaining elements into their social media marketing. Entertaining services directly impact consumers’ emotional value, subsequently influencing positive word-of-mouth intentions and repurchase behavior (Pihlström and Brush, 2008). Moreover, consumers’ perceived entertainment value of brand products and marketing content positively shapes their experiential evaluation of the brand, leading to higher brand loyalty (Lou and Xie, 2021). Similarly, Godey et al. (2016) found that social media marketing activities generate positive emotional attitudes in consumers, significantly influencing outcomes such as brand preferences and loyalty. Xu and Hu (2022) also identified perceived emotional value as a mediating variable in the relationship between stimulus variables and consumer brand loyalty. Therefore, this study proposes the following hypotheses:

*H6b*: Perceived emotional value for DTC brand consumers significantly mediates the impact of product features on loyalty.

*H7b*: Perceived emotional value for DTC brand consumers significantly mediates the impact of cost-effectiveness on loyalty.

*H8b*: Perceived emotional value for DTC brand consumers significantly mediates the impact of multi-channel integration on loyalty.

*H9b*: Perceived emotional value for DTC brand consumers significantly mediates the impact of social media marketing on loyalty.

**Figure 1:**
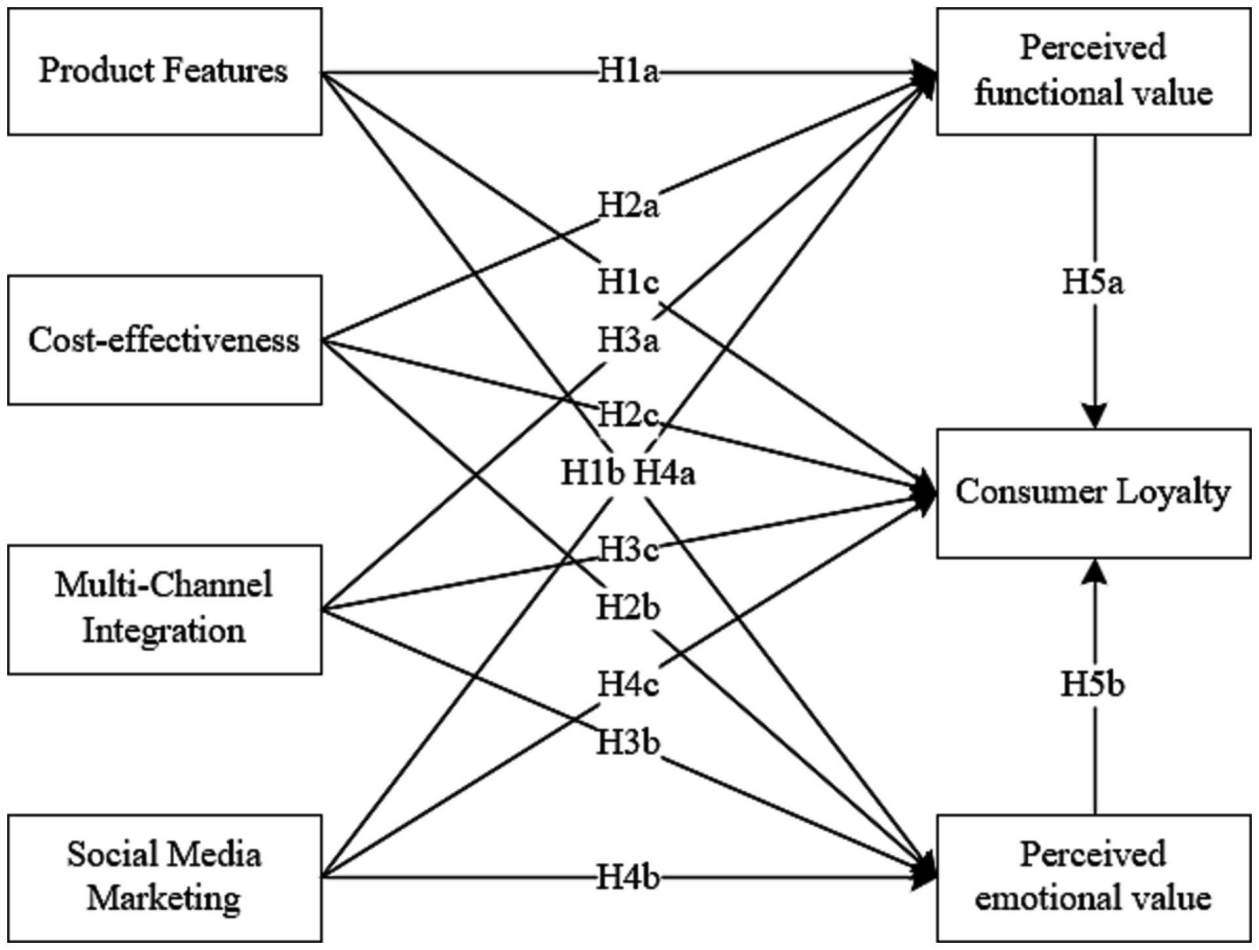
Research model based on the SOR theory.

Based on the research hypotheses and arguments presented above, the research model is constructed as shown in Figure 1.

## 4 Research methodology

### 4.1 Questionnaire design and data collection

#### 4.1.1 Scale sources

The measurement items for each latent variable in this study are based on existing mature scales and adapted to the DTC marketing model. The scale proposed by Kim et al. (2021) is used to measure brand uniqueness, brand innovativeness, and cost-effectiveness. Product features and cost-effectiveness are assessed using seven and three items, respectively. A scale by Sousa and Voss (2006) is adapted to measure the four dimensions of multichannel integration, with three items for each dimension. A social media marketing scale developed by Alalwan (2018) is adapted and uses 10 items. The measurement of perceived functional value and perceived emotional value is adapted from the well-recognized scale developed by Sweeney and Soutar (2001), and assesses the values using three items and four items, respectively. Finally, the measurement scale for loyalty is sourced from Lee and Kim (2010), comprising three items. The specific measurement scale is presented in Table 1.

**Table 1:** Variable measurement items and sources.

Latent variable			Measurement items	Source
Product features	Uniqueness	UQ1	I believe DTC brands are exceptionally unique.	Kim et al. (2021)
		UQ2	DTC brands are unparalleled.	
		UQ3	DTC brands are genuinely exceptional.	
	Innovativeness	IV1	DTC brands exhibit dynamism.	
		IV2	DTC brands consistently introduce new products and shape market trends.	
		IV3	DTC brands continually generate fresh ideas.	
		IV4	DTC brands embody forward-thinking.	
Cost-effectiveness		CE1	Considering the price, I believe DTC brand products (services) are good.	Kim et al. (2021)
		CE2	Regarding product quality, I find the prices of DTC brands to be good.	
		CE3	DTC brands offer reasonable quality and prices.	
Multi-channel Integration	Service construct transparency	TRA1	Prior to making a purchase, I am acquainted with both the online and offline stores of DTC brands.	Sousa and Voss (2006)
		TRA2	I am well-informed about the online and offline stores of DTC brands.	
		TRA3	I know how to utilize the unique features of DTC brand’s online and offline stores to fulfill my requirements.	
	Channel choice freedom	BRE1	DTC brands facilitate online ordering with offline store pickup.	
		BRE2	DTC brands accommodate online ordering with offline store returns/exchanges or repairs.	
		BRE3	The offline stores of DTC brands offer after-sales services for products ordered online.	
	Content consistency	CON1	The product information across DTC brand’s online and offline stores is consistent.	
		CON2	The price information between DTC brand’s online and offline stores is consistent.	
		CON3	The promotional information between DTC brand’s online and offline stores is consistent.	
	Process consistency	PRO1	The service image across DTC brand’s online and offline stores is consistent.	
		PRO2	The level of customer service across DTC brand’s online and offline stores is consistent.	
		PRO3	The timeliness of service remains consistent across DTC brand’s online and offline stores.	
Social media marketing	Informativeness	IM1	The marketing information shared by DTC brands on social media is a valuable source of product information.	Alalwan (2018)
		IM2	The marketing information published by DTC brands on social media is a reliable source of up-to-date product information.	
		IM3	The marketing information published by DTC brands on social media is a convenient source of product information.	
		IM4	The marketing information published by DTC brands on social media provides timely product information.	
	Relevance	RV1	The marketing information shared by DTC brands on social media is relevant to my interests.	
		RV2	I believe the marketing information published by DTC brands on social media aligns with my interests.	
		RV3	Overall, I find the marketing information published by DTC brands on social media to be suitable for me.	
	Interactivity	IA1	The marketing information shared by DTC brands on social media gives me the impression that the company values listening to customers’ feedback.	
		IA2	The marketing information published by DTC brands on social media provides customers with convenient feedback opportunities.	
		IA3	The marketing information published by DTC brands on social media promotes two-way communication between users and the company.	
Perceived functional value		PFV1	I believe DTC brand products are of good quality.	Sweeney and Soutar (2001)
		PFV2	I believe DTC brand products offer better value for money compared to products in the same price range.	
		PFV3	I consider DTC brand products to be reliable in terms of quality.	
Perceived emotional value		PAV1	I really like DTC brands.	
		PAV2	DTC brands uplift my mood.	
		PAV3	I experience happiness when engaging with DTC brands.	
		PAV4	DTC brands resonate with my personality.	
Consumer loyalty		CL1	I would recommend DTC brand stores to my friends.	Lee and Kim (2010)
		CL2	I am likely to spend more time shopping at DTC brand stores.	
		CL3	In the future, I am more likely to shop at DTC brand stores.	

#### 4.1.2 Questionnaire design

To ensure the feasibility of the questionnaire, a preliminary survey was conducted on a small sample of DTC brand consumers. A reliability and validity test was conducted on the pre-collected small-sample data. In the pre-survey stage, internal consistency analysis was employed to measure reliability, represented by the Cronbach’s alpha coefficient. Validity of the scale was examined using indicators such as KMO value, Bartlett’s sphericity test value, and factor loadings. After testing, all indicators met the standard thresholds, indicating good reliability and validity of the scale. The final version of the questionnaire, examining consumers’ perceptions, attitudes, and concerns about DTC brands, consisted of three parts.

First, the questionnaire introduces the DTC marketing model and related concepts to investigate consumers’ understanding of DTC brands and their purchasing behavior. Second, using a five-point Likert scale, consumers are asked to rate both their perceptions and actual behaviors when purchasing DTC brand products. The scale ranges from 1 to 5, representing “strongly disagree, ““disagree, ““neutral, ““agree, “and “strongly agree, “respectively. This section of the questionnaire examines seven research variables with a total of 42 measurement items. Finally, personal information including gender, birth year, education, occupation, and income, is surveyed.

#### 4.1.3 Data collection

The questionnaire was created, distributed, and the responses collected using the Chinese professional survey platform “Wenjuanxing.” Participants were recruited through a combination of online social media posts and in-person requests at offline consumer locations for passersby to scan QR codes. The survey commenced on August 22, 2023 and concluded on September 28, 2023. A total of 600 questionnaires were distributed. After screening and eliminating invalid questionnaires that were incomplete, had inconsistent response times and content, or presented conflicting personal information, 514 valid questionnaires were obtained. The questionnaire validity rate was 85.67%. Descriptive demographic statistics of the survey sample are presented in Table 2.

**Table 2:** Demographic distribution of sample [*N* = 514].

Demographics	Category	Frequency	Percentage%
Gender	Male	231	44.9%
	Female	283	55.1%
Education	High school or below	44	8.6%
	College	129	25.1%
	Bachelor’s degree	235	45.7%
	Master’s degree or above	106	20.6%
Occupation	Student	157	30.5%
	Employees of Enterprises	178	34.6%
	Government/institution	87	16.9%
	self-employed	63	12.3%
	Others	29	5.6%
Year of Birth	1997–2012	295	57.4%
	1981–1996	166	32.3%
	1965–1980	38	7.4%
	1946–1964	15	2.9%
Individual income	≤¥3,000	192	37.4%
	¥3,000–¥8,000	166	32.3%
	¥8,000–¥20,000	109	21.2%
	≥¥20,000	47	9.1%

### 4.2 Data analysis and model testing

SPSS 22 software is an effective tool for conducting preliminary statistical analysis and it was used in this study for reliability testing and exploratory factor analysis, laying the groundwork for subsequent structural equation modeling. Amos 24 software facilitates the construction and validation of complex structural equation models, exploring relationships between variables through path analysis and causal relationship testing. When addressing complex research questions and conducting multivariate analyses, structural equation modeling is considered to be more suitable than traditional regression analysis (Byrne, 2001). Consequently, in this study, the software was employed for validity testing, common method bias testing, and constructing structural equation models.

#### 4.2.1 Reliability test

Cronbach’s α coefficient assesses the internal consistency among different items in a questionnaire, confirming whether they measure the same underlying concept. It is commonly used in data validation to represent the reliability of a scale, with a threshold greater than 0.6 required for meeting recognized standards, and values above 0.7 indicating high reliability (Souza et al., 2017). In this study, Cronbach’s α values for all 13 latent variables are 0.8 or above, indicating satisfactory reliability. Specific results of the reliability test are presented in Table 3.

**Table 3:** Results of reliability and convergent validity tests.

Variables		Items	Cronbach’s α	Factor loading	AVE	CR
Product features	Uniqueness	UQ1	0.882	0.852	0.714	0.882
		UQ2		0.828		
		UQ3		0.854		
	Innovativeness	IV1	0.922	0.862	0.748	0.922
		IV2		0.866		
		IV3		0.874		
		IV4		0.857		
Cost-effectiveness		CE1	0.882	0.836	0.725	0.888
		CE2		0.856		
		CE3		0.845		
Multi-channel integration	Service construct transparency	TRA1	0.883	0.865	0.754	0.902
		TRA2		0.852		
		TRA3		0.822		
	Channel choice freedom	BRE1	0.892	0.856	0.734	0.892
		BRE2		0.862		
		BRE3		0.852		
	Content consistency	CON1	0.885	0.829	0.722	0.886
		CON2		0.874		
		CON3		0.845		
	Process consistency	PRO1	0.866	0.824	0.682	0.866
		PRO2		0.824		
		PRO3		0.830		
Social media marketing	Informativeness	IM1	0.902	0.847	0.698	0.903
		IM2		0.847		
		IM3		0.827		
		IM4		0.821		
	Relevance	RV1	0.885	0.837	0.721	0.886
		RV2		0.836		
		RV3		0.874		
	Interactivity	IA1	0.882	0.846	0.712	0.881
		IA2		0.836		
		IA3		0.850		
Perceived functional value		PFV1	0.842	0.821	0.641	0.842
		PFV2		0.821		
		PFV3		0.758		
Perceived emotional value		PAV1	0.901	0.840	0.694	0.901
		PAV2		0.836		
		PAV3		0.829		
		PAV4		0.828		
Consumer loyalty		CL1	0.879	0.843	0.707	0.879
		CL2		0.832		
		CL3		0.848		

#### 4.2.2 Validity test

##### 4.2.2.1 Structural validity test

The validity test assessed three aspects—structural validity, convergent validity, and discriminant validity. Prior to factor analysis, the Kaiser–Meyer–Olkin (KMO) test and Bartlett’s sphericity test were conducted. The KMO value is a statistical indicator measuring the suitability of factor analysis. Generally, a KMO value greater than 0.6 is considered acceptable. The Bartlett’s sphericity test determines if the data are suitable for factor analysis. If the *p-*value is less than 0.05, it indicates that the data are not completely correlated (Shrestha, 2021). The obtained KMO value is 0.897, exceeding the threshold of 0.7. The Bartlett’s sphericity test yields an approximate chi-square value of 14522.25 with 861 degrees of freedom and a significance *p-*value of 0, meeting the criterion of *p-*value less than 0.01. This indicates that the data are suitable for factor analysis.

Using SPSS 22, an exploratory factor analysis is conducted on the 42 items of the scale. Principal component analysis is employed for factor extraction based on eigenvalues greater than 1, utilizing the maximum variance method for factor rotation. The maximum convergence iteration is set to 25 times. Thirteen factors with eigenvalues greater than 1 are extracted, with each factor’s item loadings exceeding 0.7. The cumulative variance contribution rate is 80.42%, far exceeding the standard threshold of 60%. This suggests that the scale in this study passes the structural validity test and is considered ideal.

##### 4.2.2.2 Convergent validity test

The assessment of convergent validity relies on three indicators: standardized factor loadings, composite reliability (CR), and average variance extracted (AVE). Standardized factor loadings measure the extent to which each observed variable influences the latent variable. Composite reliability reflects the reliability of internal consistency within the measurement model, ranging from 0 to 1, with higher values indicating greater reliability. Typically, a CR greater than 0.7 is required. AVE measures the proportion of variance in the observed variables relative to the measurement error for the latent variable. A value exceeding 0.5 suggests a good explanatory ability of the model (Fornell and Larcker, 1981). Using Amos 24, a confirmatory factor analysis is conducted for the 13 latent variables. The standardized factor loadings for all variables in this study are above 0.7, CR values exceed 0.8, and AVE values reach 0.6 or higher—all meeting the validation criteria. These statistics indicate that each latent variable adequately represents its corresponding items, demonstrating good convergent validity. Detailed results of the convergent validity test are presented in Table 3.

##### 4.2.2.3 Discriminant validity test

The discriminant validity is assessed by analyzing the Pearson correlation coefficients between different latent variables. Pearson correlation coefficient is a measure of the strength and direction of a linear relationship between two variables. Analyzing these coefficients determines whether the latent variables possess unique explanatory power. If the Pearson correlation coefficients between latent variables are low, it indicates weak correlation and high discriminant validity (Sedgwick, 2012). As shown in Table 4, the correlation coefficients between the 13 latent variables are generally at or below 0.5, all falling below the standard threshold of 0.85. Moreover, they are all lower than the square root of their respective AVE, indicating that the correlation between latent variables is lower than the correlation between observed variables. This suggests that the latent variables exhibit certain correlations while maintaining sufficient distinctiveness, confirming the discriminant validity of the measurement model. Detailed results of descriptive statistics, correlation coefficients, and discriminant validity are presented in Table 4.

**Table 4:** Descriptive statistics, correlation coefficients, and discriminant validity of variables.

	Mean	SD	CL	PAV	PFV	IA	RV	IM	PRO	CON	BRE	TRA	CE	IV	UQ
CL	3.43	1.05	0.84												
PAV	3.41	1.04	0.56**	0.83											
PFV	3.46	0.98	0.53**	0.50**	0.8										
IA	3.39	1.09	0.39**	0.38**	0.33**	0.84									
RV	3.42	1.07	0.38**	0.37**	0.37**	0.62**	0.85								
IM	3.30	1.04	0.44**	0.39**	0.36**	0.58**	0.60**	0.84							
PRO	3.40	1.03	0.34**	0.24**	0.29**	0.25**	0.30**	0.25**	0.83						
CON	3.37	1.09	0.28**	0.27**	0.32**	0.23**	0.26**	0.26**	0.60**	0.85					
BRE	3.27	1.11	0.20**	0.15**	0.23**	0.18**	0.21**	0.24**	0.50**	0.61**	0.86				
TRA	3.37	1.09	0.20**	0.18**	0.27**	0.22**	0.23**	0.19**	0.51**	0.65**	0.58**	0.87			
CE	3.32	1.08	0.35**	0.31**	0.24**	0.31**	0.27**	0.22**	0.15**	0.21**	0.14**	0.08**	0.85		
IV	3.21	1.14	0.14**	0.15**	0.11**	0.07**	0.12**	0.15**	0.18**	0.20**	0.15**	0.24**	0.03**	0.86	
UQ	3.23	1.12	0.26**	0.31**	0.20**	0.23**	0.18**	0.26**	0.16**	0.22**	0.14**	0.17**	0.15**	0.61**	0.84

CL, consumer loyalty; PAV, perceived emotional value; PFV, perceived functional value; IA, interactivity; RV, relevance; IM; informativeness; PRO, process consistency; CON, content consistency; BRE, channel choice freedom; TRA, service construct transparency; CE, cost effectiveness; IV, innovativeness; UQ, uniqueness. The lower triangular matrix represents the correlation coefficients between variables, while the diagonal values represent the square root of Average Variance Extracted (AVE) for each corresponding variable. **p < 0.01.

#### 4.2.3 Common method bias test

Due to potential similarities that may lead to artificial covariance arising from the survey environment, data sources, and survey timing, it is crucial to rigorously examine the possibility of common method bias. A robust method, confirmatory factor analysis (CFA) with the addition of a common method factor, was used to assess common method bias. Model fit is evaluated using the indices X^2^/df, RMSEA, CFI, and GFI. The X^2^/df reflects the degree of difference between actual data and the model; a smaller X^2^/df indicates better model-data fit. RMSEA gauges the average level of error in the model, with values below 0.05 generally considered indicative of good model fit. CFI, GFI, and similar indices represent fit, with values ranging from 0 to 1, where values closer to 1 suggest better fit. The initial model (M1) constructed in this study exhibits favorable fit indices, with X^2^/df = 1.161, RMSEA = 0.018 (meeting the standard of ≤0.08), and CFI = 0.992, along with other GFI indices above 0.9 (Hu and Bentler, 1999). When introducing a common method factor to create model M2, the fit indices remain comparable to those of the original model M1, with X^2^/df = 1.114, RMSEA = 0.015, and non-significant changes in other fit indices, all below 0.1 and less than 0.03. These results indicate that the addition of a common method factor does not significantly improve the fit of model M2 compared to the original model M1. Therefore, there is no apparent common method bias in this measurement. Detailed fit indices are presented in Table 5.

**Table 5:** Model fit statistics.

Fit indices	X^2^/df	RMSEA	GFI	AGFI	CFI	IFI	TLI	NFI	RFI
Evaluation criterion	<3	<0.05	>0.9	>0.9	>0.9	>0.9	>0.9	>0.9	>0.9
Model 1	1.161	0.018	0.928	0.912	0.992	0.992	0.990	0.942	0.933
Model 2	1.114	0.015	0.935	0.916	0.994	0.994	0.993	0.948	0.936
Fit discrepancy	0.047	0.003	0.007	0.004	0.002	0.002	0.003	0.006	0.003

## 5 Results

### 5.1 Structural equation model (SEM) and path testing

**Figure 2:**
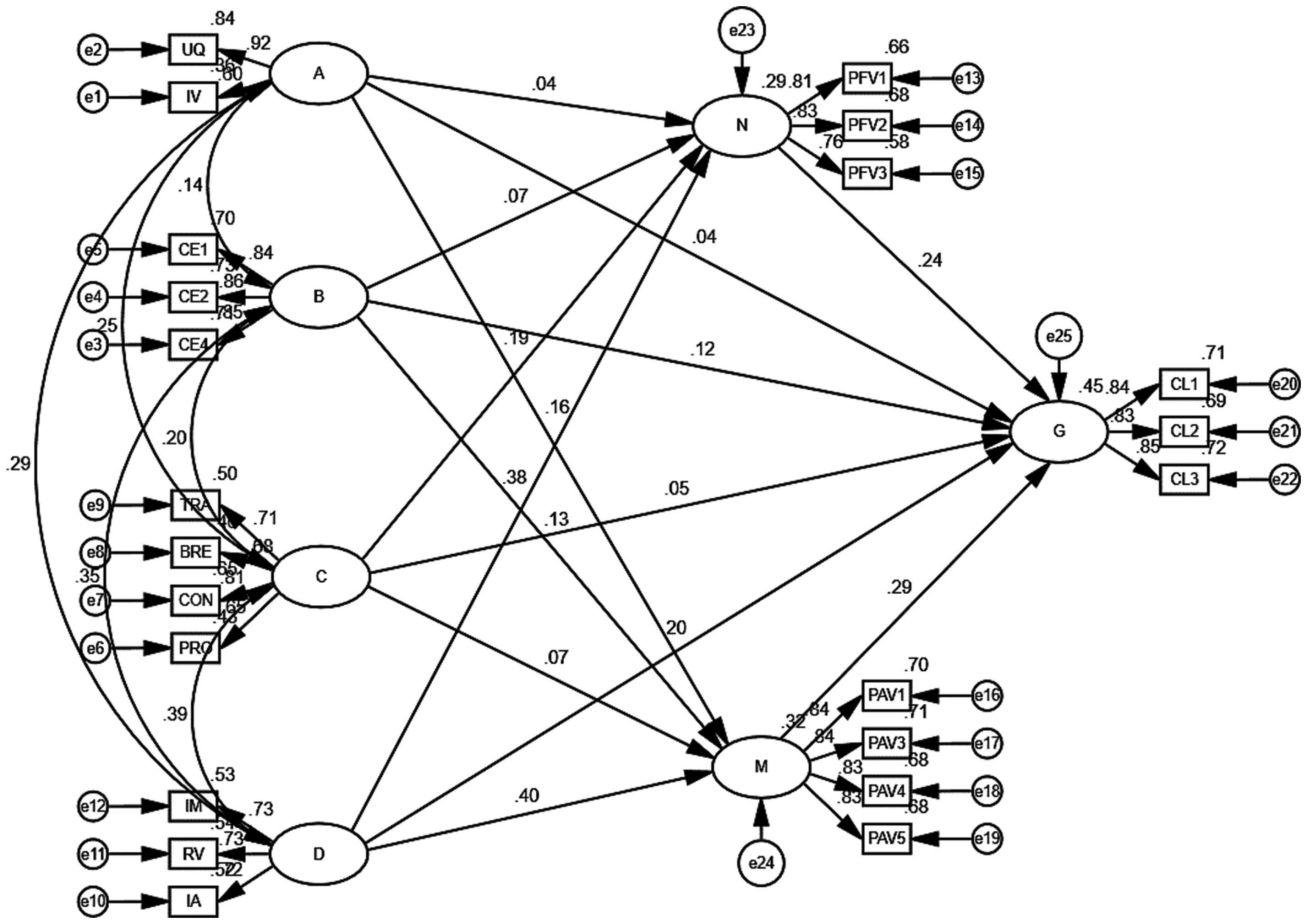
Structural equation model diagram. A, Product features; B, Cost effectiveness; C, Multi-channel integration; D, Social media marketing; N, Perceived functional value; M, Perceived emotional value; G, Consumer loyalty.

The SEM depicting the influence relationships among the factors of DTC marketing model characteristics, perceived functional value, perceived emotional value, and consumer loyalty is constructed using Amos 24 software. To facilitate model construction and analysis, an item parceling strategy is applied to the observed variables of first-order latent variables with second-order latent variables. The finalized SEM is presented in Figure 2. In addition, the specific numerical values of the path analysis results are presented in Table 6. The significance level is represented by the Critical Ratio (C.R.) value—where an absolute value greater than 1.96 indicates that the path is significant at the 0.05 level—suggesting that the two variables influence each other significantly.

**Table 6:** Results of path analysis.

Hypothesized paths	*β*	S.E.	C.R.	*p*
Product features → Perceived functional value	0.040	0.069	0.789	0.430
Product features → Perceived emotional value	0.158	0.074	3.229	**
Cost effectiveness → Perceived functional value	0.071	0.048	1.399	0.162
Cost effectiveness → Perceived emotional value	0.135	0.051	2.811	**
Multi-channel integration → Perceived functional value	0.195	0.078	3.450	***
Multi-channel integration → Perceived emotional value	0.067	0.081	1.257	0.209
Social media marketing → Perceived functional value	0.384	0.078	5.870	***
Social media marketing → Perceived emotional value	0.400	0.082	6.431	***
Product features → Consumer loyalty	0.041	0.065	0.906	0.365
Cost effectiveness → Consumer loyalty	0.118	0.046	2.661	**
Multi-channel integration → Consumer loyalty	0.049	0.072	1.011	0.312
Social media marketing → Consumer loyalty	0.205	0.080	3.257	**
Perceived functional value → Consumer loyalty	0.244	0.058	4.553	***
Perceived emotional value → Consumer loyalty	0.285	0.051	5.373	***

*β*, standardized path coefficients; S.E., approximate standard errors; C.R., critical ratio; ***p < 0.001, **p < 0.01, *p < 0.05.

### 5.2 Direct effect hypothesis testing

Based on the direct effect testing results presented in Table 7, the following nine direct path hypotheses were validated: H1b (*β* = 0.158, *p* < 0.01), H2b (*β* = 0.135, *p* < 0.01), H2c (*β* = 0.118, *p* < 0.01), H3a (*β* = 0.195, *p* < 0.001), H4a (*β* = 0.384, *p* < 0.001), H4b (*β* = 0.400, *p* < 0.001), H4c (*β* = 0.205, *p* < 0.01), H5a (*β* = 0.244, *p* < 0.001), H5b (*β* = 0.285, *p* < 0.001). However, the following five hypotheses were not supported: H1a (*β* = 0.040, *p* > 0.05), H1c (*β* = 0.071, *p* > 0.05), H2a (*β* = 0.071, *p* > 0.05), H3b (*β* = 0.067, *p* > 0.05), H3c (*β* = 0.049, *p* > 0.05). The results of the direct effect hypothesis testing indicate that within the DTC marketing model characteristics, multi-channel integration and social media marketing significantly positively influence consumers’ perceived functional value. In addition, product features and cost-effectiveness have a significant positive impact on perceived emotional value. Additionally, cost-effectiveness, social media marketing, and both perceived functional and emotional value have a direct positive impact on consumer loyalty.

**Table 7:** Hypothesis testing results for direct effects.

Research hypotheses		Results
H1a	Product features → Perceived functional value	Not Supported
H1b	Product features → Perceived emotional value	Supported
H1c	Product features → Consumer loyalty	Not Supported
H2a	Cost effectiveness → Perceived functional value	Not Supported
H2b	Cost effectiveness → Perceived emotional value	Supported
H2c	Cost effectiveness → Consumer loyalty	Supported
H3a	Multi-channel integration → Perceived functional value	Supported
H3b	Multi-channel integration → Perceived emotional value	Not Supported
H3c	Multi-channel integration → Consumer loyalty	Not Supported
H4a	Social media marketing → Perceived functional value	Supported
H4b	Social media marketing → Perceived emotional value	Supported
H4c	Social media marketing → Consumer loyalty	Supported
H5a	Perceived functional value → Consumer loyalty	Supported
H5b	Perceived emotional value → Consumer loyalty	Supported

The test results generally support the hypotheses proposed in this study. Hypotheses that were not supported may be attributed to several factors. The DTC marketing model is in its early development stage, and due to cost and market limitations, it may not effectively address consumers’ perceived functional and emotional values (Naz, 2022). Additionally, modern consumers with high demands for uniqueness and innovation may prefer diverse innovative products or brands, and their loyalty can be influenced by various factors (Naim, 2023). Furthermore, the study’s sample size is relatively small, and the survey of DTC brand consumers may not be fully representative. Moreover, traditional brands currently still dominate the market, and people lack awareness of DTC brands and their marketing models, which may have impacted the survey results (Hansen et al., 2013). Therefore, although certain characteristics of DTC marketing models may be necessary conditions for eliciting favorable emotions or perceived usefulness from consumers, they are insufficient to directly increase consumer loyalty.

### 5.3 Mediation hypothesis testing

A bootstrap testing method was employed to examine the mediating effects present in this study (Hayes and Scharkow, 2013). The results are presented in Table 8. Based on the reported effect values, H6a, H7a, and H8b were not supported, meaning that these three paths have non-significant mediating effects. Conversely, H6b, H7b, H8a, H9a, and H9b were supported, indicating significant mediating effects for these five paths. Additionally, due to the absence of direct effects between product features and loyalty—as well as multi-channel integration and loyalty—there is a full mediating effect of product features and multi-channel integration on loyalty. Further, because there are direct effects between cost-effectiveness and loyalty, as well as social media marketing and loyalty, there is only a partial mediating effect of cost-effectiveness and social media marketing on loyalty. Therefore, the positive impact of different characteristics of the DTC marketing model on loyalty operates through various aspects of perceived value. Product features and cost-effectiveness can influence consumer loyalty by affecting perceived emotional value, while multi-channel integration positively influences consumer loyalty through the mediation of perceived functional value. Additionally, social media marketing has a positive impact on loyalty through both functional and emotional perceived values.

**Table 8:** Hypothesis testing results of standardized bootstrap mediation effects.

Path		Effect size	SE	Bias-corrected 95%CI			Percentile 95%CI			Result
				Lower	Upper	*P*	Lower	Upper	*P*	
H6a	Product features → Perceived functional value → Consumer loyalty	0.01	0.02	−0.01	0.05	0.39	−0.02	0.04	0.54	Not supported
H6b	Product features → Perceived emotional value → Consumer loyalty	0.05	0.02	0.01	0.10	0.01	0.01	0.09	0.02	Supported
H7a	Cost effectiveness → Perceived functional value → Consumer loyalty	0.02	0.02	−0.01	0.06	0.16	−0.01	0.05	0.22	Not supported
H7b	Cost effectiveness → Perceived emotional value → Consumer loyalty	0.04	0.02	0.01	0.09	0.02	0.00	0.08	0.03	Supported
H8a	Multi-channel integration → Perceived functional value → Consumer loyalty	0.05	0.02	0.02	0.10	0.00	0.02	0.09	0.00	Supported
H8b	Multi-channel integration → Perceived emotional value → Consumer loyalty	0.02	0.02	−0.01	0.06	0.20	−0.01	0.06	0.24	Not supported
H9a	Social media marketing → Perceived functional value → Consumer loyalty	0.09	0.03	0.05	0.17	0.00	0.04	0.16	0.00	Supported
H9b	Social media marketing → Perceived emotional value → Consumer loyalty	0.11	0.03	0.06	0.19	0.00	0.06	0.18	0.00	Supported

### 5.4 Impact weights of the DTC marketing model’s characteristics

Due to the varying degrees of influence of the four characteristics of the DTC marketing model on consumer loyalty, it is essential to further explore the importance of different characteristics in eliciting loyalty. The impact weights of the key factors were therefore analyzed. The total path coefficient method was employed to calculate the overall path coefficients for product features, cost-effectiveness, multi-channel integration, and social media marketing. A larger total path coefficient indicates a greater weight of influence for the respective characteristic factor. The computation utilized the standardized path coefficients (*β* values) listed in Table 6. The total path coefficient is the sum of the direct path coefficients from the independent variable to the dependent variable and the total indirect path coefficients. The total indirect path coefficient is the sum of the products of all segmental path coefficients. The specific results are presented in Table 9. The impact weights of the DTC marketing model characteristics on loyalty are ordered as follows: Social Media Marketing (0.415) > Cost-Effectiveness (0.174) > Multi-Channel Integration (0.116) > Product Features (0.096). This ordering indicates that social media marketing is the most advantageous and crucial strategic factor in the DTC marketing model for eliciting consumer loyalty. The other three factors also play important roles in influencing loyalty to varying degrees, making them indispensable advantages within the DTC marketing model.

**Table 9:** Path coefficients of DTC marketing model characteristics factors.

Latent variable		Perceived functional value	Perceived emotional value	Consumer loyalty
Product features	Direct path	0.040	0.158	0.041
	Indirect path			0.055
	Total path	0.040	0.158	0.096
Cost effectiveness	Direct path	0.071	0.135	0.118
	Indirect path			0.056
	Total path	0.071	0.135	0.174
Multi-channel integration	Direct path	0.195	0.067	0.049
	Indirect path			0.067
	Total path	0.195	0.067	0.116
Social media marketing	Direct path	0.384	0.400	0.205
	Indirect path			0.210
	Total path	0.384	0.400	0.415

**Figure 3:**
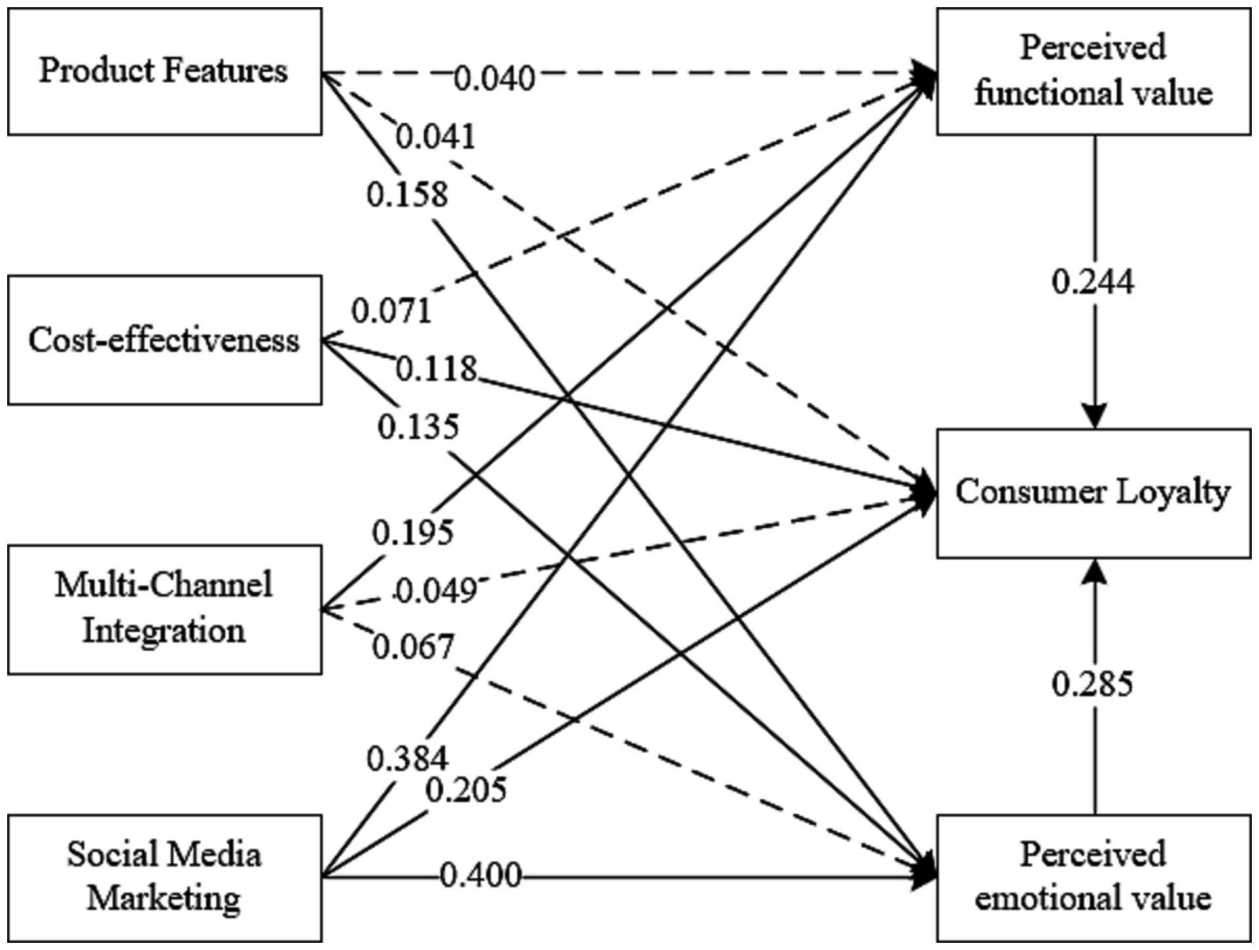
Revised research model and path diagram. Solid lines represent paths that passed the test, dashed lines indicate paths that did not pass the test, and numerical values on the paths denote the path coefficients.

In summary, by constructing an SEM to examine the direct effects, mediating effects, and variable weights, the initial theoretical model was refined based on the test results, as illustrated in Figure 3.

## 6 Conclusion and discussion

### 6.1 Key findings

This study explored the relationship between DTC marketing model characteristics in the digital intermediary era and consumer loyalty. The key findings are as follows: (1) In the digital intermediary era, the factors influencing consumer loyalty in the DTC marketing model manifest in the aspects of product, price, channel, and promotion, represented by product features, cost-effectiveness, multi-channel integration, and social media marketing. These factors align with the consumption characteristics of DTC brand consumer groups dominated by Millennials and Generation Z. (2) Cost-effectiveness and social media marketing directly impact DTC brand consumer loyalty. Additionally, product features and cost-effectiveness influence loyalty through the perception of emotional value, multi-channel integration positively affects loyalty through perceived functional value, and social media marketing information positively influences loyalty through both perceived functional and emotional values. The mediating effect of product features and multi-channel integration on loyalty is complete, while the mediating effect of cost-effectiveness and social media marketing on loyalty is partial. (3) Different characteristic factors have varying weights and degrees of impact on consumer loyalty. Social media marketing has the most significant impact, while product features have the least influence.

Based on the research findings, the following optimization recommendations are proposed for enterprises and brand marketing strategies:

- Strengthen social media marketing and enhance interactive social media marketing experiences. Social media serves as an effective tool for direct consumer engagement. Enterprises and brands should strengthen social media marketing strategies, collaborate closely with social media platforms, and publish compelling content, including unique product showcases, user stories, and brand culture to attract consumers in the digital intermediary era, such as Millennials and Generation Z.
- Optimize product features to meet consumer demands for functional innovation and personalization. Brands should continuously improve product features to ensure alignment with the needs and expectations of target consumers. Through ongoing market research and analysis of consumer feedback, understand their expectations for the brand, focus on perceived value, and integrate functional and emotional value into the product design and development process.
- Reduce information asymmetry and enhance cost transparency. In the post-pandemic era, with economic slowdown and consumer downgrading, Brands should emphasize communicating the cost-effectiveness of products, ensuring reasonable product pricing, and highlight the balance between price and quality. They should aim to align cost management measures with conveying product value, allowing consumers to perceive value for money. This approach will maintain positive perceptions of product value and brand care, enhancing customer loyalty.
- Integrate and coordinate multiple channels to convey a consistent value proposition. In practice, DTC brands can achieve close coordination and consistency between online and offline sales channels through the synergistic collaboration of multiple channels. Brands should ensure that consumers can experience both the functional and emotional value of products throughout the shopping process. They should also facilitate easy switching between channels while maintaining consistent brand awareness across different channels, increasing consumer brand stickiness.

### 6.2 Theoretical contributions

This study makes theoretical contributions to academic fields such as marketing and consumer brand loyalty by investigating the relationship between the emerging DTC marketing model and loyalty. It holds profound significance for expanding the theoretical framework in the field of marketing. First, by delving into the DTC marketing model in the digital intermediary era, this study unveils how this innovative marketing model shapes consumer loyalty under the influence of different characteristic factors. As technology continues to advance, the dynamic interactions among consumers and brands are undergoing significant changes. Understanding the impact of these changes on consumer–brand relationships is crucial for building more precise and targeted marketing strategies.

Second, analyzing the mechanisms through which DTC marketing model characteristic factors influence consumer loyalty can reveal the psychology and motivations behind consumer behavior. This not only provides businesses with deeper market insights but also offers new theoretical perspectives for academia on consumer behavior and emotional connections. Third, this study describes the impact of digital transformation on brand construction and maintenance. It contributes to enriching the theoretical framework in the field of marketing, offering new insights for the formulation of marketing strategies.

### 6.3 Practical implications

This study can be used as a base to improve several practices and behaviors. First, with the rapid development of digital technology, the DTC marketing model has become a trend in numerous brand transformations. This investigation of how characteristic factors influence consumer loyalty provides guidance for emerging DTC brands and offers direction for businesses to formulate more targeted market strategies Second, the findings assist existing DTC brands in identifying issues, pinpointing pain points, and optimizing their operations and management. In the design and operational processes, consideration should be given to fostering positive interactions between the brand and consumers, providing a unique shopping experience, and enhancing user engagement and loyalty. Third, this study can help traditional brands update and reconstruct their marketing models to adapt to the marketing environment of the digital intermediary era, aligning with the trends in digital marketing development. Lastly, the discussion contained herein can be used to raise awareness among government and regulatory bodies regarding the construction and development of DTC brands. It can also provide policymakers advice on brand support, management, and the operation of digital intermediary platforms.

### 6.4 Limitations and future research

This study has certain limitations. The sample size is small, and the sample scope is narrow, primarily focusing on Chinese consumers. Additionally, due to limited relevant literature and data, the exploration of characteristic factors in the DTC marketing model may not be exhaustive. Furthermore, the study did not categorize brands, and consumers may have different concerns regarding different types of brands. Therefore, in future research, employing big-data research methods and focusing the research on one specific category may provide a comprehensive and targeted investigation into DTC marketing models and consumer behavior. Further aspects such as consumer privacy, data security, and trust, can also be examined in future studies.

## Data availability statement

The original contributions presented in the study are included in the article/Supplementary material, further inquiries can be directed to the corresponding author.

## Ethics statement

Ethical approval was not required in compliance with national and institutional regulations. The studies were carried out in accordance with local laws and institutional requirements, and participants provided written informed consent to take part in the study.

## Author contributions

WM: Writing – original draft. YY: Writing – original draft.
